# Global analysis of gene expression in response to L-Cysteine deprivation in the anaerobic protozoan parasite *Entamoeba histolytica*

**DOI:** 10.1186/1471-2164-12-275

**Published:** 2011-05-31

**Authors:** Afzal Husain, Ghulam Jeelani, Dan Sato, Tomoyoshi Nozaki

**Affiliations:** 1 Department of Parasitology, National Institute of Infectious Diseases, 1-23-1 Toyama, Shinjuku, Tokyo 162-8640, Japan; 2 Department of Parasitology, Graduate School of Medicine, Gunma University, Maebashi 371-8511, Japan; 3 Department of Biochemistry and Integrative Medical Biology, School of Medicine, Keio University, Shinjuku, Tokyo 160-8582, Japan; 4 Institute for Advanced Biosciences, Keio University, Tsuruoka, Yamagata 997-0052, Japan; 5 Graduate School of Life and Environmental Sciences, University of Tsukuba,1-1-1 Tennodai, Tsukuba, Ibaraki 305-8572, Japan

## Abstract

**Background:**

*Entamoeba histolytica*, an enteric protozoan parasite, causes amebic colitis and extra intestinal abscesses in millions of inhabitants of endemic areas. *E. histolytica *completely lacks glutathione metabolism but possesses L-cysteine as the principle low molecular weight thiol. L-Cysteine is essential for the structure, stability, and various protein functions, including catalysis, electron transfer, redox regulation, nitrogen fixation, and sensing for regulatory processes. Recently, we demonstrated that in *E. histolytica*, L-cysteine regulates various metabolic pathways including energy, amino acid, and phospholipid metabolism.

**Results:**

In this study, employing custom-made Affymetrix microarrays, we performed time course (3, 6, 12, 24, and 48 h) gene expression analysis upon L-cysteine deprivation. We identified that out of 9,327 genes represented on the array, 290 genes encoding proteins with functions in metabolism, signalling, DNA/RNA regulation, electron transport, stress response, membrane transport, vesicular trafficking/secretion, and cytoskeleton were differentially expressed (≥3 fold) at one or more time points upon L-cysteine deprivation. Approximately 60% of these modulated genes encoded proteins of no known function and annotated as hypothetical proteins. We also attempted further functional analysis of some of the most highly modulated genes by L-cysteine depletion.

**Conclusions:**

To our surprise, L-cysteine depletion caused only limited changes in the expression of genes involved in sulfur-containing amino acid metabolism and oxidative stress defense. In contrast, we observed significant changes in the expression of several genes encoding iron sulfur flavoproteins, a major facilitator super-family transporter, regulator of nonsense transcripts, NADPH-dependent oxido-reductase, short chain dehydrogenase, acetyltransferases, and various other genes involved in diverse cellular functions. This study represents the first genome-wide analysis of transcriptional changes induced by L-cysteine deprivation in protozoan parasites, and in eukaryotic organisms where L-cysteine represents the major intracellular thiol.

## Background

L-Cysteine, a sulfur-containing amino acid (SAA), is ubiquitous in virtually all living organisms from bacteria to higher eukaryotes, and plays an essential role in the various cellular processes including stability, structure, regulation of catalytic activity, and posttranslational modification for various proteins. Due to the ability of its thiol group to undergo redox reactions, L-cysteine has antioxidant properties, and is used for the biosynthesis of glutathione, which is found in humans as well as other organisms. In addition, L-cysteine is also essential for the synthesis of trypanothione, coenzyme A, hypotaurine, taurine as well as ubiquitous iron-sulfur (Fe-S) clusters, which are involved in electron transfer, redox regulation, nitrogen fixation, and sensing for regulatory processes [1].

*Entamoeba histolytica*, an enteric protozoan parasite, causes amebic colitis and extra intestinal abscesses in millions of inhabitants of endemic areas, and responsible for thousands of deaths annually [2]. The trophozoites of *E*. *histolytica *primarily reside in the anaerobic environment of the colonic lumen, but are exposed to various reactive oxygen and nitrogen species (ROS and RNS) during tissue invasion, metastasis, and extra intestinal propagation [2,3]. *E. histolytica *lacks most of the components of the eukaryotic oxidative stress defence system including catalase, peroxidase, glutathione, and glutathione-recycling enzymes. However, it possesses alternative mechanisms for detoxification of the reactive oxygen and nitrogen species. The alternative mechanisms are most likely to involve superoxide dismutase (SOD), peroxiredoxin, flavodiiron proteins (FDPs), and reducing agents (thiols), especially L-cysteine [4-6]

Among a number of metabolic peculiarities, metabolism of SAAs in *E. histolytica *is distinct from that of its mammalian host in a variety of aspects. First, it lacks both forward and reverse trans-sulfuration pathways and thus is unable to interconvert L-methionine and L-cysteine [6] Second, it possesses methionine γ-lyase (MGL) which degrades L-methionine, L-homocysteine, and L-cysteine [7-9]. Third, *E. histolytica *possesses enzymes for the *de novo *S-methylcysteine/L-cysteine biosynthesis [10-12]. The S-methylcysteine/L-cysteine biosynthetic pathway involves serine acetyltransferase (SAT, EC2.3.1.30) that catalyzes acetyl CoA-dependent acetylation of the side chain hydroxyl group of L-serine to form O-acetylserine (OAS) [13]. Subsequently, cysteine synthase [(CS; OAS (thiol) lyase; EC4.2.99.8)] catalyzes the reaction of OAS with methanethiol or sulfide to produce S-methylcysteine or L-cysteine, respectively. Recombinant amebic CS isotypes possess both S-methylcysteine and L-cysteine synthesizing activities *in vitro*. However, our recent *in vivo *study [12] revealed that CS isotypes are primarily involved in the synthesis of SMC, but not of L-cysteine. Since, this pathway is not involved in the synthesis of L-cysteine, *in vitro *cultivation of amebic trophozoites requires high concentrations of L-cysteine, and this requirement can not be replaced by other thiols [14]. In *E. histolytica*, L-cysteine is required for the growth, attachment, survival, and protection from oxidative stress [14,15].

All prokaryotic and eukaryotic cells are known to have an ability to restructure their transcriptomes in order to adapt to the environmental conditions by sensing the endogenous level of various metabolites. Small-molecule metabolites, including amino acids, nucleotides, and carbohydrates have been shown to regulate the expression of large number of genes at the transcriptional and post-transcriptional levels [16]. In addition, intracellular redox determined by various metabolites has also been demonstrated to be an important regulator to gene expression [16].

In most eukaryotes, glutathione is the major thiol, and L-cysteine levels are maintained many fold lower than that of glutathione [17]. However, *E. histolytica *completely lacks glutathione metabolism and relies on L-cysteine as a major redox buffer [5, 6, and 8]. Therefore, *E. histolytica *represents an excellent model to study the effect of L-cysteine deprivation on gene expression and cellular metabolism. Our recent metabolomic study demonstrated that in *E. histolytica*, L-cysteine regulates various metabolic pathways, including energy, amino acid, and phospholipid metabolism [12]. In this study we performed DNA microarray analysis of gene expression in *E. histolytica *cultured in L-cysteine-deprived conditions. We found that the expression of a large number of genes was modulated in response to the L-cysteine deprivation.

## Results and Discussions

### L-Cysteine deprivation induces global changes in the gene expression

**Figure 1:**
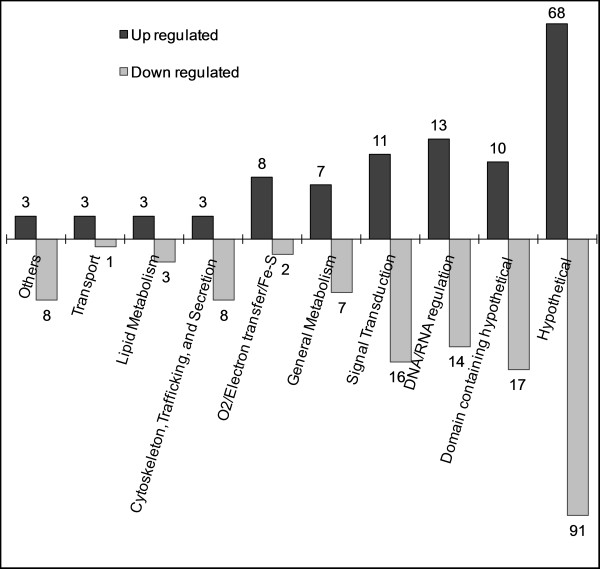
**Functional distribution of differentially expressed genes upon L-cysteine deprivation**. Genes were grouped according to putative functions inferred from the respective annotations. The exact number of changed transcripts is shown beside each column.

To better understand the role of L-cysteine in transcriptional regulation of gene expression in *E. histolytica*, we performed time course analysis of genome wide gene expression upon L-cysteine deprivation, using a custom-made Affymetrix microarray representing 9,327 of *E. histolytica *genes. We identified 290 genes (3.1%) modulated by at least 3 fold (p-value < 0.05) at one or more time points in response to L-cysteine deprivation (Additional file 1). Out of them, 129 genes were up-regulated and 167 genes were down-regulated, while 6 genes showed both up- and down-regulation depending upon the time points (Tables 1 and 2; Additional files 2 and 3). Out of the 129 up-regulated genes, 51 genes (40%) were assigned with putative biological functions, namely signalling, general metabolism, lipid metabolism, DNA/RNA regulation, electron transport, stress response, transport, and trafficking/secretion/cytoskeleton (Figure 1). The remaining 78 genes (60%) were categorized into genes encoding either hypothetical proteins without (68) or with known conserved domain(s) (10). A total of 167 genes were down regulated by ≥3 fold at one or more time points upon L-cysteine deprivation, 108 (65%) of which encode hypothetical proteins or hypothetical proteins containing conserved domain(s), whereas remaining 59 genes (35%) encode proteins with putative biological functions (Figure 1).

**Table 1:** List of most highly induced genes upon L-cysteine deprivation

Probe set ID	Accession numbers	Common Names	Basal Expression (log_2_)	3 h	6 h	12 h	24 h	48 h	p value
EHI_173950_at	XM_647419	Major facilitator superfamily (MFS) transporter	6.11	+ 4.1	+ 14.6	+ 10.0	+ 4.7	+ 2.6	3.0E-07
EHI_138480_at	XM_650038	Iron-sulfur flavoprotein, putative	8.40	+ 3.6	+ 6.8	+ 9.8	+ 5.4	+ 4.2	4.5E-08
EHI_025710_at	XM_644279	Iron-sulfur flavoprotein, putative	7.17	+ 3.6	+ 5.4	+ 8.7	+ 5.2	+ 3.9	1.0E-06
13.m00350_at	XM_651312	Hypothetical protein	2.73	- 1.0	+ 2.4	+ 7.8	+ 5.4	+ 6.5	2.4E-04
EHI_176810_at	XM_644746	Hypothetical protein	3.53	+ 4.8	+ 8.8	+ 6.0	+ 1.2	- 1.3	4.6E-04
EHI_130490_at	XM_643338	Hypothetical protein	7.42	+ 1.7	+ 1.9	+ 5.8	+ 3.5	+ 1.7	1.8E-05
EHI_091050_at	XM_645468	Zinc finger protein, putative (IBR superfamily)	5.39	+ 2.9	+ 9.1	+ 5.7	- 1.4	- 1.7	1.3E-06
EHI_080280_at	XM_644430	Glu6-phosphate N-acetyltransferase. putative	3.79	+ 8.6	+ 6.2	+ 5.5	+ 1.3	- 1.3	3.3E-05
EHI_032670_s_at	XM_645799	Iron sulfur flavoprotein like, putative	7.47	+ 1.5	+ 3.5	+ 5.4	+ 3.9	+ 4.0	3.4E-06
870.m00013_x_at	XM_642792	Hypothetical protein	2.54	+ 3.4	+ 6.4	+ 5.0	+ 1.3	+ 1.9	6.5E-04
EHI_096770_at	XM_650580	Acetyltransferase, putative	7.01	+ 1.9	+ 3.4	+ 4.8	+ 3.7	+ 4.2	4.9E-05
EHI_137260_at	XM_647486	Hypothetical protein	2.97	+ 3.7	+ 4.4	+ 4.8	+ 2.4	+ 2.6	2.0E-03
65.m00145_x_at	XM_648920	Hypothetical protein	3.08	+ 1.2	+ 3.3	+ 4.7	+ 1.1	- 1.6	6.1E-06
EHI_062300_at	XM_645096	Hypothetical protein	4.10	+ 1.7	+ 1.9	+ 4.7	+ 2.7	+ 2.1	1.7E-03
337.m00049_x_at	XM_644075	Hypothetical protein	2.48	+ 2.9	+ 5.2	+ 4.5	+ 1.7	+ 1.0	1.5E-03
EHI_148740_at	XM_001913345	Hypothetical protein	8.19	+ 1.9	+ 2.7	+ 4.4	+ 1.7	+ 1.3	1.0E-06
79.m00141_x_at	XM_648476	Hypothetical protein	2.90	+ 2.0	+ 3.1	+ 4.4	+ 1.7	+ 1.2	2.4E-02
EHI_189190_x_at	XM_644225	Hypothetical protein	3.25	+ 1.7	+ 4.8	+ 4.4	- 1.0	- 1.9	4.3E-05
EHI_039720_at	XM_642957	Hypothetical protein	2.53	- 1.0	+ 2.1	+ 4.4	+ 3.3	+ 1.4	1.6E-05
EHI_051040_s_at	XM_647890	Hypothetical protein	6.82	+ 1.5	+ 2.7	+ 4.4	+ 2.5	+ 2.8	1.9E-03
EHI_139080_at	XM_643428	Longevity-assurance family protein	5.38	- 2.3	+ 1.2	+ 4.4	+ 2.3	+ 2.0	4.4E-06
EHI_067230_x_at	XM_647567	Hypothetical protein	6.18	+ 9.7	+ 13.6	+ 4.2	- 1.3	- 1.1	1.4E-07
EHI_055680_at	XM_646949	Heat shock protein, Hsp20 family, putative	5.63	+ 2.0	+ 4.6	+ 4.2	+ 1.6	+ 1.2	1.4E-03
EHI_086500_s_at	XM_646060	Short chain dehydrogenase	4.86	+ 6.1	+ 8.1	+ 4.2	+ 1.1	- 1.4	1.2E-04
EHI_148970_s_at	XM_652477	Regulator of nonsense transcripts, putative	9.86	+ 5.9	- 1.0	- 4.1	- 5.1	- 9.7	1.5E-07
EHI_139090_at	XM_643429	Hypothetical protein	5.95	+ 1.6	+ 1.9	+ 4.1	+ 1.3	- 1.5	6.2E-05
167.m00129_at	XM_646319	Hypothetical protein	3.36	+ 2.4	+ 4.8	+ 4.0	+ 2.0	+ 1.9	1.6E-03
EHI_110840_s_at	XM_649191	Regulator of nonsense transcripts, putative	9.73	+ 6.5	+ 1.0	- 4.0	- 4.6	- 8.7	1.2E-07
EHI_178130_at	XM_646412	Hypothetical protein	5.09	+ 2.0	+ 3.2	+ 4.0	+ 2.4	+ 2.8	5.9E-05
EHI_110370_at	XM_651955	Hypothetical protein	4.52	- 1.3	+ 1.3	+ 3.9	+ 2.6	+ 4.1	2.0E-04
EHI_070810_x_at	XM_649317	Regulator of nonsense transcripts, putative	4.76	+ 7.4	- 1.1	- 3.9	- 4.3	- 4.8	1.6E-05
EHI_004990_at	XM_647768	Ankyrin, putative	6.30	+ 2.0	+ 4.3	+ 3.8	+ 2.4	+ 3.0	5.1E-05
EHI_023330_at	XM_650547	Hypothetical protein	9.16	+ 6.5	+ 1.4	- 3.6	- 7.1	- 8.3	8.2E-07
EHI_005160_s_at	XM_647757	Hypothetical protein	2.94	+ 2.9	+ 4.5	+ 3.4	+ 1.6	+ 1.2	6.7E-03
EHI_028940_at	XM_647571	Hypothetical protein	8.44	+ 6.1	+ 6.5	+ 3.4	- 1.0	- 1.2	1.4E-07
EHI_033240_x_at	XM_645809	Riboflavin kinase/FAD synthetase, putative	6.61	+ 1.4	+ 4.6	+ 3.4	+ 3.0	+ 2.6	8.6E-07
493.m00030_x_at	XM_643175	Hypothetical protein	8.73	+ 3.9	+ 5.2	+ 3.3	- 1.8	- 1.9	1.4E-07
15.m00356_at	XM_651173	Hypothetical protein	7.03	+ 3.4	+ 4.2	+ 3.3	+ 1.7	+ 2.1	1.8E-03
EHI_110480_at	XM_651901	Hypothetical protein	4.51	+ 2.3	+ 4.2	+ 3.3	+ 2.9	+ 1.7	1.5E-04
EHI_140620_x_at	XM_645555	Hypothetical protein	2.32	+ 2.1	+ 5.9	+ 3.2	+ 1.3	+ 1.2	2.3E-02
EHI_136430_at	XM_650171	Hypothetical protein	4.08	+ 7.5	+ 7.8	+ 3.2	- 2.3	+ 1.0	4.1E-06
373.m00052_s_at	XM_643804	Hypothetical protein	6.53	+ 4.1	+ 2.0	+ 3.1	+ 1.0	- 1.1	7.9E-04
EHI_141030_at	XM_649510	DNA methyltransferase, putative	5.97	+ 2.4	+ 1.7	+ 2.3	+ 3.9	+ 4.1	3.6E-05
EHI_178520_at	XM_647939	Regulator of nonsense transcripts, putative	6.30	+ 6.2	- 1.4	- 2.1	- 1.9	- 1.4	8.7E-06
EHI_142270_at	XM_648119	Hypothetical protein	8.27	+ 3.7	+ 4.1	+ 2.0	- 1.0	- 1.1	2.9E-04
EHI_014340_at	XM_649988	Hypothetical protein	6.45	+ 2.7	+ 5.2	+ 1.9	+ 1.1	- 1.1	4.9E-05
EHI_064440_at	XM_648922	Hypothetical protein	5.03	- 1.5	- 1.2	+ 1.7	+ 2.5	+ 4.0	2.0E-03
EHI_120930_s_at	XM_649257	Protein kinase domain containing protein	2.61	+ 6.8	- 1.0	+ 1.5	+ 2.3	+ 1.8	2.4E-03
637.m00013_s_at	XM_642916	Regulator of nonsense transcripts, putative	2.92	+ 6.6	- 1.2	- 1.5	- 1.5	- 1.1	3.6E-05
EHI_031640_at	XM_648447	Hypothetical protein	6.98	+ 2.3	- 3.1	+ 1.5	+ 3.6	+ 5.8	1.8E-06
EHI_103640_at	XM_643960	Protein kinase domain containing protein	3.10	+ 5.2	- 1.0	- 1.4	+ 1.4	+ 1.2	5.6E-04
EHI_014910_s_at	XM_001914428	Hypothetical protein	2.99	+ 6.0	+ 1.5	- 1.3	+ 1.4	- 1.2	2.7E-04
EHI_038910_at	XM_651787	Hypothetical protein	2.57	+ 5.3	+ 2.3	+ 1.2	+ 1.6	- 1.0	5.1E-03
EHI_190460_at	XM_646352	Amino acid transporter, putative	3.11	- 1.2	- 1.4	+ 1.2	+ 2.2	+ 3.9	2.9E-03
EHI_084710_at	XM_650002	Hypothetical protein	4.27	+ 5.3	+ 2.3	+ 1.1	+ 2.2	+ 1.2	5.1E-04
EHI_054680_at	XM_646972	Hypothetical protein	3.09	- 1.2	- 1.4	- 1.1	+ 2.7	+ 5.9	4.8E-04
50.m00196_s_at	XM_649450	Hypothetical protein	4.95	+ 4.2	- 1.5	- 1.1	+ 1.2	- 1.4	9.0E-05
EHI_046040_s_at	XM_645992	Hypothetical protein	5.08	+ 6.5	+ 1.5	- 1.1	- 1.0	- 1.6	1.1E-05

The probe set IDs, accession numbers, common names, basal expressions, fold changes, and p values of most highly induced genes upon L-cysteine deprivation are shown.

**Table 2:** List of most highly down-regulated genes upon L-cysteine deprivation

Probe set ID	Accession numbers	Common Names	Basal Expression (log_2_)	3h	6h	12h	24h	48h	p value
72.m00179_at	XM_648717	Hypothetical protein	8.8	+ 1.1	+1.4	- 2.8	- 15.3	- 22.8	2.5E-06
EHI_045340_s_at	XM_648481	NADPH-dependent oxidoreductase (EhNO2)	10.6	- 1.9	-1.9	- 3.0	- 10.6	- 8.3	1.4E-07
EHI_023330_at	XM_650547	Hypothetical protein	9.2	+ 6.5	+1.4	- 3.6	- 7.1	- 8.3	8.2E-07
EHI_148970_s_at	XM_652477	Regulator of nonsense transcripts, putative	9.9	+ 5.9	-1.0	- 4.1	- 5.1	- 9.7	1.5E-07
EHI_110840_s_at	XM_649191	Regulator of nonsense transcripts, putative	9.7	+ 6.5	+1.0	- 4.0	- 4.6	- 8.7	1.2E-07
EHI_070810_x_at	XM_649317	Regulator of nonsense transcripts, putative	4.8	+ 7.4	-1.1	- 3.9	- 4.3	- 4.8	1.6E-05
EHI_049960_at	XM_651359	Hypothetical protein	7.8	+ 2.4	-1.6	- 5.1	- 3.3	- 2.9	5.1E-07
EHI_182260_s_at	XM_001914319	Cysteine protease, putative	7.8	- 1.7	-3.2	- 2.5	- 3.2	- 6.3	2.7E-05
EHI_052890_at	XM_645369	Hypothetical protein	9.0	- 1.1	-6.3	- 11.1	- 3.0	- 2.6	3.5E-05
EHI_077280_s_at	XM_649853	Leucine rich repeat protein, BspA family	8.4	- 5.4	-6.7	- 2.2	- 2.7	- 1.1	2.5E-03
EHI_178790_at	XM_651153	Hypothetical protein	4.7	- 1.7	-5.1	- 1.6	+ 2.5	+ 3.0	1.2E-04
EHI_094060_s_at	XM_001913553	Actin binding protein, putative	9.2	+ 1.3	-3.5	- 5.0	- 2.5	- 3.2	1.8E-03
371.m00031_s_at	XM_643815	Leucine rich repeat protein	8.3	- 4.3	-3.2	- 2.4	- 2.4	- 1.4	1.5E-02
EHI_049570_at	XM_650791	RhoGAP domain containing protein	6.9	+ 2.1	-2.3	- 5.0	- 2.3	- 2.6	8.7E-04
EHI_130710_at	XM_644068	Myb-like DNA-binding protein	6.3	+ 1.3	-1.9	- 4.4	- 2.2	- 1.9	1.6E-05
EHI_197440_at	XM_646593	Hypothetical protein	10.6	+ 1.5	-2.7	- 4.1	- 1.9	- 2.6	1.0E-03
330.m00075_x_at	XM_644126	Hypothetical protein	6.9	+ 1.1	+1.0	- 3.3	+ 1.8	- 7.2	1.9E-03
EHI_148650_at	XM_652381	Leucine rich repeat/phosphatase domain	7.2	- 2.4	-4.5	- 2.9	- 1.7	- 1.6	1.2E-03
		containing protein							
EHI_050660_at	XM_651502	Hypothetical protein	7.3	+ 1.7	-3.7	- 4.4	- 1.7	- 1.6	8.1E-04
EHI_044890_at	XM_652115	Helicase, putative	5.6	- 1.0	-4.3	- 2.3	- 1.6	- 1.4	6.5E-03
49.m00187_x_at	XM_649517	Fatty acid elongase, putative	4.8	+ 1.3	-5.6	- 5.4	- 1.6	- 1.7	3.3E-05
EHI_094780_s_at	XM_001914195	Pescadillo homolog, putative	5.7	+ 1.4	-2.9	- 4.0	- 1.6	- 2.1	8.5E-03
260.m00059_s_at	XM_644821	Phospholipase D like protein	4.4	+ 1.7	-3.6	- 4.2	- 1.6	- 3.0	2.1E-03
EHI_140530_at	XM_649099	Hypothetical protein	4.9	+ 1.4	-2.3	- 4.5	+ 1.6	+ 1.3	1.1E-04
EHI_145850_at	XM_650629	Hypothetical protein	4.9	+ 1.1	-3.2	- 5.8	- 1.5	- 2.0	2.3E-03
EHI_046630_at	XM_645444	Rho family GTPase	6.0	- 1.1	-4.4	- 4.4	+ 1.5	- 1.2	2.2E-04
EHI_187100_at	XM_651384	Hypothetical protein	6.9	- 1.3	-4.4	- 2.4	- 1.5	- 1.1	2.5E-06
849.m00008_s_at	XM_642804	Leucine rich repeat protein	7.9	- 5.1	-3.5	- 2.1	- 1.5	- 1.1	1.4E-02
EHI_009840_s_at	XM_652013	Hypothetical protein	11.1	- 4.3	-1.2	+ 1.4	+ 1.5	+ 1.6	4.0E-07
EHI_039330_at	XM_648669	Hypothetical protein	9.3	+ 1.1	-4.3	- 2.9	- 1.5	- 1.4	2.6E-02
266.m00066_s_at	XM_644755	Hypothetical protein	7.1	+ 1.6	+1.0	- 1.3	+ 1.4	- 6.6	1.3E-05
EHI_029500_s_at	XM_644980	Hypothetical protein	6.9	+ 1.7	-1.0	- 1.4	+ 1.4	- 7.4	1.6E-04
216.m00082_x_at	XM_645403	Hypothetical protein	6.0	- 2.3	-4.1	- 3.9	- 1.4	- 1.8	4.4E-04
EHI_196770_s_at	XM_001914173	Leucine rich repeat protein	8.2	- 3.4	-4.6	- 2.1	- 1.3	+ 1.0	2.9E-02
EHI_034590_s_at	XM_001914026	Hypothetical protein	7.1	+ 1.2	-1.2	- 1.4	+ 1.3	- 6.4	5.2E-05
EHI_107660_at	XM_644692	Hypothetical conserved, protein	9.1	- 5.7	-2.0	+ 1.1	+ 1.3	+ 1.7	1.1E-06
207.m00059_x_at	XM_645533	Actinin-like protein, putative	5.4	+ 1.7	-5.4	- 2.4	- 1.3	- 1.6	6.4E-03
EHI_182540_at	XM_651612	Hypothetical protein	6.3	+ 1.0	-3.2	- 4.1	+ 1.3	- 1.0	5.4E-04
EHI_010130_at	XM_651999	Hypothetical protein	6.6	- 9.5	-5.3	- 1.3	- 1.3	- 1.1	4.0E-06
EHI_018030_s_at	XM_001914259	Hypothetical protein	7.1	+ 1.4	+1.0	- 1.1	+ 1.3	- 11.0	4.3E-05
EHI_020830_s_at	XM_001913952	Hypothetical protein	9.4	+ 1.4	-1.3	- 1.2	+ 1.3	- 9.9	1.4E-05
EHI_002240_s_at	XM_645752	Hypothetical protein	7.1	+ 1.4	-1.0	- 1.2	+ 1.2	- 6.6	1.9E-04
EHI_196760_s_at	XM_643708	Hypothetical protein	9.4	+ 1.5	-1.3	- 1.2	+ 1.2	- 8.4	1.0E-05
EHI_037700_s_at	XM_643865	Hypothetical protein	7.1	+ 1.4	-1.1	- 1.3	+ 1.2	- 7.0	3.3E-05
EHI_189540_x_at	XM_645163	Hypothetical protein	5.0	+ 1.5	-4.1	- 2.4	- 1.2	- 2.1	9.9E-03
EHI_196720_s_at	XM_643106	Hypothetical protein	7.1	+ 1.5	-1.1	- 1.3	+ 1.2	- 10.1	2.8E-04
375.m00058_s_at	XM_643788	Hypothetical protein	7.1	+ 1.5	-1.1	- 1.3	+ 1.2	- 6.3	2.6E-04
194.m00101_s_at	XM_645779	Hypothetical protein	7.2	+ 1.4	-1.1	- 1.4	+ 1.2	- 6.9	8.0E-05
372.m00048_s_at	XM_643812	Protein kinase domain containing protein	6.0	+ 1.1	-4.0	- 4.0	+ 1.1	- 1.1	1.8E-04
EHI_144150_s_at	XM_001914451	Hypothetical protein	6.3	+ 1.8	-1.6	- 2.0	+ 1.1	- 13.2	1.5E-04
EHI_020840_s_at	XM_001913953	Hypothetical protein	7.4	+ 1.7	-1.4	- 1.9	- 1.1	- 18.9	4.1E-05
EHI_103840_at	XM_648260	DNA repair protein, putative	6.1	+ 1.2	-3.1	- 4.3	+ 1.0	- 1.1	3.1E-03
506.m00025_s_at	XM_643137	Hypothetical protein	8.3	+ 1.1	-1.0	- 1.9	- 1.0	- 6.8	9.9E-04
390.m00061_s_at	XM_643707	Hypothetical protein	7.4	+ 1.7	-1.5	- 1.7	- 1.0	- 12.6	1.1E-05
EHI_145330_s_at	XM_001913871	Hypothetical protein	6.5	+ 1.2	-1.1	- 1.8	- 1.0	- 6.5	5.1E-05
EHI_099250_at	XM_649200	Hypothetical protein	6.3	+ 1.2	-1.9	- 4.0	- 1.0	- 1.3	2.1E-04
EHI_196770_s_at	XM_001914173	Hypothetical protein	7.4	+ 1.8	-1.4	- 1.5	- 1.0	- 17.1	2.7E-05
190.m00086_s_at	XM_645857	Hypothetical protein	8.4	+ 1.0	-1.1	- 2.0	- 1.0	-6.4	1.1E-03

The probe set IDs, accession numbers, common names, basal expressions, fold changes, and p values of most highly down-regulated genes upon L-cysteine deprivation are shown.

To verify the data obtained by Affymetrix-based microarray, we performed quantitative RT-PCR on five genes: two each from significantly up- (EHI_173950 and EHI_138480) and down-regulated genes (EHI_045340 and EHI_052890), respectively, and one invariant gene (EHI_056690), based on Affymetrix analysis. The results of qRT-PCR agreed well with the microarray data for all five transcripts tested (Table 3). The modulated genes were grouped into broad categories, based on the protein BLAST at NCBI and InterProScan at EMBL, and discussed below (Figure 1).

**Table 3:** Verification of the microarray data by qRT-PCR

Common Name	Accession Number	Fold Change				
		
		3 h	6 h	12 h	24 h	48 h
Major fascilitator superfamily (MFS) transporter	XM_647419	2.0	12.8	7.2	4.2	2.3
		
		(4.1)	(14.6)	(9.9)	(4.7)	(2.6)

Iron sulfur flavoprotein (ISF)	XM_650038	1.8	4.4	14.9	5.5	2.8
		
		(3.6)	(6.8)	(9.8)	(5.4)	(4.2)

NADPH-dependent oxidoreductase (EhNO2)	XM_648481	-3.0	-3.0	-6.0	-8.4	-7.8
		
		(-1.9)	(-1.9)	(-3.0)	(-10.6)	(-8.3)

Hypothetical protein	XM_645369	-2.2	-6.4	-12.9	-3.4	-2.7
		
		(-1.1)	(-6.3)	(-11.1)	(-3.0)	(-2.6)

RNA polymerase II 15-kDa subunit	XM_643999	1.0	-1.2	1.1	-1.3	-1.3
		
		(1.4)	(1.2)	(1.2)	(1.1)	(1.2)

The common names, accession numbers, and fold changes of the selected genes upon L-cysteine deprivation are shown. The upper values are the fold changes in the expression obtained from qRT-PCR. The corresponding fold changes in the expression values obtained from Affymetrix analysis are shown in brackets.

### Effect of L-cysteine deprivation on SAA metabolism

**Figure 2:**
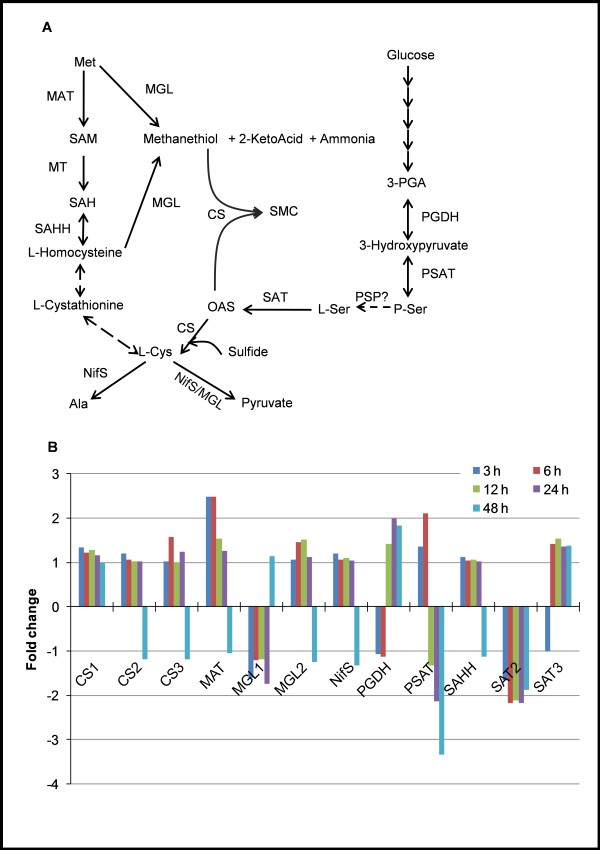
**Effect of L-cysteine deprivation on expression of the genes involved in sulfur-containing amino acid metabolism**. ***A***) General scheme of sulfur-containing amino acid metabolism in *E. histolytica*. Abbreviations: CS, cysteine synthase; SAT, serine O-acetyltransferase; PGDH, phosphoglycerate dehydrogenase; MGL, methionine γ-lyase; MAT, methionine adenosyl-transferase; MT, methyl-transferase; SAHH, S-adenosylhomocysteine hydrolase; NifS, cysteine desulfurase; PSAT, phosphoserine aminotransferase; PSP, phosphoserine phosphatase; OAS; O-acetylserine; SMC, S-methylcysteine; SAH, S-adenosylhomocysteine; SAM, S-adenosyltransferase; P-Ser, O-phosphoserine; 3-PGA, 3-phosphoglycerate. ***B***) Modulation of transcripts encoding enzymes involved in sulfur containing amino acid metabolism. Gene IDs: CS1, EHI_171750; CS2, EHI_160930; CS3, EHI_060340; MAT, 70.m00173; MGL1, EHI_144610; MGL2, EHI_142250; NifS; EHI_136380; PGDH, EHI_060860; PSAT, EHI_026360; SAHH, EHI_068250; SAT2, EHI_021570; SAT3, EHI_153430.

To further explore the role of L-cysteine in the regulation of expression of genes involved in SAA metabolism and associated pathways, we investigated their expression upon L-cysteine deprivation. As shown in Figure 2A, most of the genes involved in SAA metabolism except phosphoserine aminotransferase (PSAT) were not modulated by >3 fold upon L-cysteine deprivation. PSAT, an enzyme that catalyzes the reversible conversion of 3-phosphohydroxypyruvate to L-phosphoserine, the second step of phosphorylated L-serine biosynthetic pathway, was down-regulated by 3.3 fold at 48 h (Figure 2B). Other genes that were slightly modulated by L-cysteine deprivation included methionine adenosyltransferase (MAT) and phosphoglycerate dehydrogenase (PGDH), which were induced by >2 fold at early (3-6 h) and late (24-48 h) time points of L-cysteine deprivation, respectively (Figure 2B). This lack of changes in the expression of genes involved in SAA metabolism might be due to their high basal expression (except CS3 and SAT2, which have relatively low expression) under normal conditions(Additional file 4). Alternatively, it may be because L-cysteine has a very limited influence on the expression of the genes involved in SAA metabolism in *E. histolytica*. However, L-cysteine has been shown to significantly modulate the metabolic flux across SAA metabolism in *E. histolytica *[12]. In contrast to *E. histolytica*, L-cysteine availability is known to have a significant influence on the expression of the genes involved in SAA metabolism in other eukaryotic cells [18]. For example, in HepG2/C3A cells, L-cysteine deprivation resulted in the induction of cysteinyl-tRNA synthetase, glutamate-cysteine ligase, L-cystine-glutamate transporter, cystathionine γ-lyase, and glutamate-cysteine ligase, and a down-regulation of 3-phosphoadenosine 5-phosphosulfate synthase and sulfite oxidase [18].

We have recently shown by metabolomic analysis that the synthesis of OAS and SMC markedly increased upon L-cysteine deprivation in *E. histolytica*. OAS in bacteria is known to regulate the genes of cysteine regulon, and increment in its level modulates the expression of the genes involved in L-cysteine and sulfide synthesis [19]. However, no such regulation of genes of cysteine biosynthetic pathway was observed in *E. histolytica*, except a 2 fold down-regulation of a gene encoding SAT2, and slight induction of a gene encoding SAT3 (Figure 2B). These results imply that L-cysteine does not significantly modulate expression of the genes involved in SAA metabolism in *E. histolytica*; however, it affects the flux of SAA metabolism by post-transcriptional or post-translational mechanisms.

### Effect of L-cysteine deprivation on the genes involved in oxidative and nitrosative stress defense

**Figure 3:**
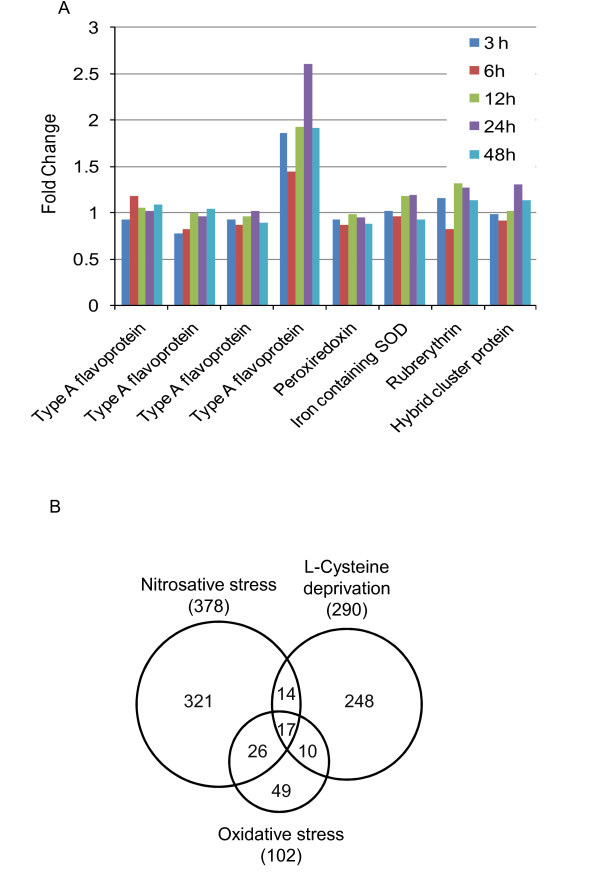
**Comparison of the *E. histolytica *genes modulated upon L-cysteine deprivation and oxidative or nitrosative stress**. ***A***) Expression kinetics upon L-cysteine deprivation of the genes previously inferred for oxidative and/or nitrosative stress defense. Gene/protein ID of enzymes are: type A flavoproteins (Flavodiiron proteins), EHI_159860, EHI_064530, EHI_096710, and EHI_129890; peroxiredoxin, EHI_145840; iron containing SOD, EHI_159160; rubrerythrin, EHI_134810; hybrid cluster protein, EHI_004600. ***B**) *A Venn diagram showing the number of overlapping genes modulated upon L-cysteine deprivation and oxidative (1 mM of H_2_O_2 _for 1 h), or nitrosative stress (200 μM of DPTA-NONOate for 1 h), as described [23].

The *E. histolytica *genome contains several genes encoding ROS and RNS detoxifying proteins, such as peroxiredoxin, rubrerythrin, hybrid-cluster protein, superoxide dismutase (SOD), and flavodiiron proteins (FDPs) [6]. FDPs are widespread in prokaryotes, and known to be involved in the reduction of oxygen and/or nitric oxide whereas peroxiredoxin, rubrerythrin, hybrid-cluster protein, and superoxide dismutase (SOD) are involved in the detoxification of H_2_O_2 _and/or superoxide radicals [20-22]. Although L-cysteine deprivation led to the increment in the level of intracellular ROS [12], the genes encoding putative ROS-and RNS-detoxifying proteins in *E. histolytica *were not significantly modulated (Figure 3A). This is consistent with the previous studies that genes encoding known ROS and RNS detoxification pathways are not modulated in response to H_2_O_2_-mediated oxidative or DPTA-NONOate-mediated nitrosative stress in *E. histolytica *[23]. The lack of induction of the genes involved in oxidative/nitrosative stress is likely due to their high baseline expression even in the absence of oxidative or nitrosative stress. While most of the known genes in the ROS and RNS detoxification pathways were not modulated, one (EHI_129890) of the four FDP genes was slightly (up to 2.6 fold) up-regulated upon L-cysteine deprivation (Figure 3A). These findings suggest that *E. histolytica *might employ other post-transcriptional or post-translational regulatory mechanisms, such as RNA transport, protein modifications, allosteric regulations, and redirection of metabolic fluxes, to cope up with the oxidative stress.

The comparison of the genes modulated in response to L-cysteine deprivation with those modulated upon oxidative or nitrosative stress showed a very limited overlap (Figure 3B). Genes modulated upon L-cysteine deprivation shared only 27 or 31 genes with those modulated by oxidative or nitrosative stress, respectively (Figure 3B). Of these shared genes, 17 genes were shared by all the three conditions, suggesting that these genes play a general (or central) role in the response against L-cysteine deprivation and oxidative/nitrosative stress. A list of these shared genes is shown in Additional file 5. Among the genes that were up-regulated by L-cysteine deprivation and oxidative/nitrosative stress were several genes encoding iron sulfur flavoproteins (ISF) (EHI_067720, EHI_025710, EHI_138480). Interestingly, ISFs were among the most highly up-regulated genes by L-cysteine deprivation. ISFs constitute a wide-spread family of redox-active proteins found predominantly in anaerobic prokaryotes [24]. They are flavin mononucleotide (FMN) cofactor, and iron-sulfur [Fe-S] clusters containing proteins with an unusually compact cysteine motif [25]. The deduced amino acid sequences of amebic ISFs also suggest the presence of this compact cysteine motif (CX2CX2CX5-7C) that is most likely involved in the ligation of [4Fe-4S] clusters [25,26].

**Figure 4:**
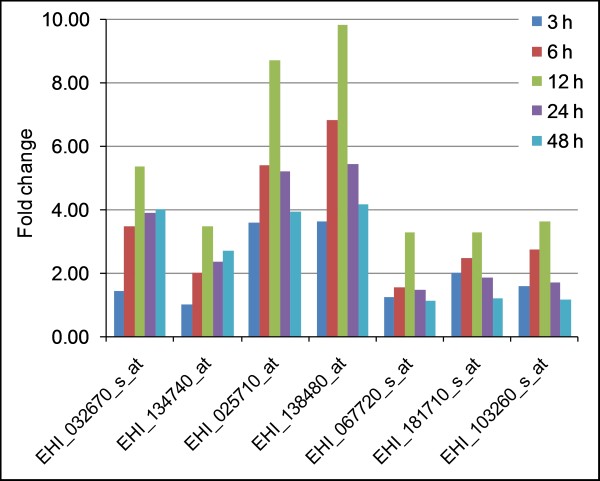
**Induction of iron sulfur flavoproteins upon L-cysteine deprivation in *E. histolytica***. Values are expressed as fold changes of the transcripts under L-cysteine-deprived conditions, relative to the trophozoites cultured in medium containing normal concentration of L-cysteine.

Iron sulfur flavoproteins belong to a novel family of proteins that are widely distributed in distantly related anaerobic prokaryotes. Interestingly, *E. histolytica *and *Trichomonas vaginalis *are the only members of the domain *Eukarya *that possess ISFs [6, 26]. There are at least 7 independent genes for ISFs in the genome of *E. histolytica*. However, the total number entries in *E. histolytica *database representing ISF genes is 13 as some of the sequences show very high mutual sequence identities (95-99%). A total of 7 probe sets representing 5 different ISF genes were up regulated ≥3 fold at one or more time points upon L-cysteine deprivation (Figure 4). Two of these ISF genes (EHI_138480 and EHI_025710) showed a maximum induction of 9.8 and 8.7 fold at 12 h of L-cysteine deprivation, respectively. The remaining probe sets were induced by only 3-6 folds upon L-cysteine deprivation. Three ISF genes, including two ISF genes highly induced upon L-cysteine deprivation (EHI_138480 and EHI_025710), were also induced by oxidative stress [23]. In contrast to their induction in response to L-cysteine deprivation or oxidative stress, two ISF genes (EHI_067720, EHI_134740) were down-regulated by 2-5 folds on day 1 and day 29 in the mouse model of intestinal amoebiasis [27]. These findings suggest that the expression of ISFs is regulated by the availability of the reactive oxygen species, and agree with the proposed function of ISFs in anaerobes in combating oxidative stress by reducing O_2 _and H_2_O_2 _to water [28]. In addition to oxidative stress, ISFs and ISF-related proteins were also induced upon the deprivation of sulfate or L-cysteine in bacteria [29].

### Effect of L-Cysteine deprivation on membrane transport

**Figure 5:**
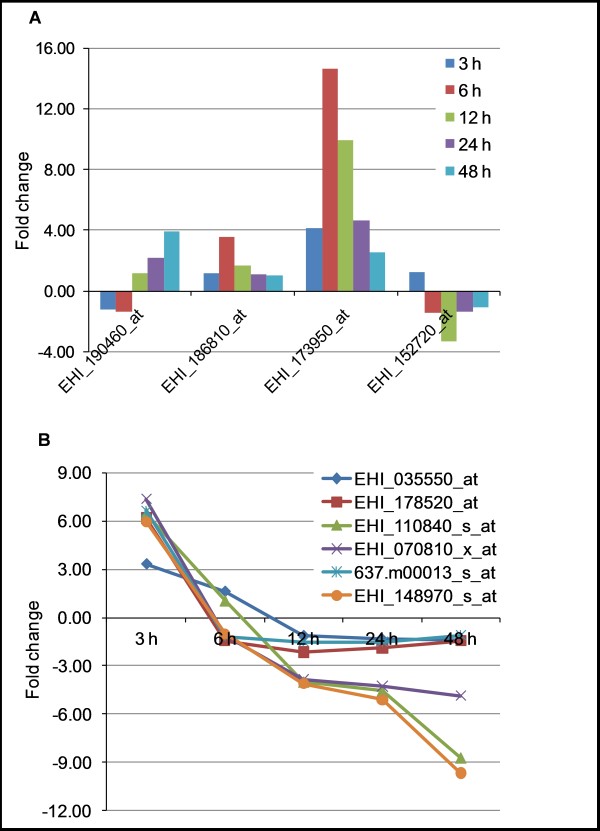
**Changes in the expression of the genes encoding membrane transporters and regulator of nonsense transcripts upon L-cysteine deprivation**. Values are expressed as fold changes in the expression of the transcripts relative to the trophozoites culture in the presence of normal concentration of L-cysteine. ***A***) Changes in the expression of the genes encoding putative transporter proteins. ***B***) Time-dependent changes in the expression of the genes encoding putative regulators of nonsense transcripts.

Adaptive response to altered environmental conditions may include a significant alteration in the gene expression of the membrane transporters that are involved in the intake or efflux of various metabolites. A total of 4 genes with putative transport functions were significantly modulated in response to the removal of L-cysteine from the culture medium (Figure 5A). Two genes (EHI_173950 and EHI_186810) encoding major facilitator super-family (MFS) transporters showed maximum induction of 14.6 (EHI_173950) and 3.5 fold (EHI_186810) at 6 h upon L-cysteine deprivation. The third gene (EHI_190460) that encodes for an amino acid transporter was also induced by 3.9 fold at 48 h, whereas the fourth gene (EHI_152720) that encodes a small conductance mechanosensitive ion channel was down-regulated by 3.7 at 12 h upon L-cysteine deprivation (Additional files 2 and 3). The increments (~2 fold) in L-serine and L-threonine levels upon L-cysteine deprivation [12] may be attributed to either increased expression of amino acid transporter or MFS transporter, or reversal of L-cysteine-mediated inhibition of their transporters.

MFS is a large superfamily of membrane transporters present ubiquitously in bacteria, archaea, and eukarya [30]. They are involved in the symport, antiport, or uniport of various substrates including sugars, phosphorylated glycolytic intermediates, amino acids, polyols, drugs, neurotransmitters, and osmolites [30]. MFS transporters from yeast and bacteria are known to be involved in the transport of the metabolites of L-cysteine biosynthetic pathway including L-cysteine and O-acetylserine [31,32]. L-Cysteine deprivation resulted in drastic increments in various metabolites such as SMC, OAS, glycerol 3-phosphate and isopropanolamine, and sharp decrements in L-cysteine and L-cystine [12]. Thus, it may be possible that these MFS transporters are involved in either intake or efflux of the metabolites modulated upon L-cysteine deprivation. As the contribution of L-cysteine biosynthetic pathway to L-cysteine synthesis is negligible, both L-cysteine and L-cystine are completely deprived upon L-cysteine deprivation. Under this condition, *E. histolytica *trophozoites may induce expression of certain high affinity L-cysteine or L-cystine transporters. The genome of *E. histolytica *contains about 24 different genes for MFS transporters [6]. However, exact substrate specificities, and physiological roles of these MFS transporters in *E. histolytica *remain to be established.

### Effect of L-cysteine deprivation on general metabolism

Recently, we demonstrated that in addition to the drastic metabolic changes in SAA metabolism, L-cysteine also regulates other metabolic pathways including phospholipid and energy metabolism [12]. However, like SAA metabolism, most of the genes involved in phospholipid or energy metabolism showed only minor changes in their expressions in response to L-cysteine deprivation. However, some transcriptional changes in the expression of genes involved in energy metabolism were noted. Genes encoding hexokinase, phosphoglycerate mutase, and malate dehydrogenase were slightly down regulated (Additional file 1). Down-regulation of these genes may partially contribute to the overall decrease in the metabolic flux across glycolysis as reported in our previous metabolomic study of L-cysteine deprivation [12].

Transcripts that showed significant induction upon L-cysteine deprivation include a gene encoding putative glucosamine 6-phosphate N-acetyltransferase (EHI_080280), which is known to be involved in chitin biosynthetic pathway (Table 1). Two other genes encoding putative acetyltransferases (103.m00159, EHI_096770) were also induced 3-5 fold upon L-cysteine deprivation (Table 1; Additional file 2). These acetyltransferases contain maltose/galactose-O-acetyltransferase domains, and are known to be involved in the acetylation of a variety of substrates such as maltose, galactose, glucosamine, glucose, and fructose. However, the exact substrate specificity, physiological relevance, and the pathways that these acetyltransferases are involved in, are not known in *E. histolytica*. EHI_096770 was also induced upon H_2_O_2_-mediated oxidative (4 fold) or DPTA-NONOate-mediated nitrosative stress (2.7 fold) in *E. histolytica *[23]. A gene encoding cyst wall specific glycoprotein Jacob was induced during the early time points (Additional file 2). Both glucosamine 6-phosphate N-acetyltransferase and glycoprotein Jacob are involved in the encystation to form a chitin cell wall. However, it is not clear why the enzymes of chitin biosynthetic pathway are induced upon L-cysteine deprivation. Because some of the components of chitin biosynthetic pathway are known to be induced by oxidative stress [33], it is possible that stress induced by L-cysteine deprivation is also responsible for their induction.

A gene encoding riboflavin kinase/FAD synthetase that is involved in the synthesis of FAD or FMN cofactors was also induced up to 4.6 fold as an early response to L-cysteine deprivation (Table 1). This may imply that there is an increase demand of FMN or FAD cofactors during L-cysteine deprivation. A gene (EHI_086500) encoding short chain dehydrogenase/reductases (SDR) was also induced up to 8 fold during early time points. SDR are NAD^+^/NADP^+^-dependent oxido-reductases, and are similar to alcohol dehydrogenases (Table 1). Recently, we demonstrated that L-cysteine deprivation led to the accumulation of isopropanolamine, and *E. histolytica *possesses a pathway for its synthesis from methylglyoxal via aminoacetone [12]. SDR may be involved in the synthesis of isopropanolamine. Further biochemical analysis is required to better understand the significance of this L-cysteine-regulated dehydrogenase in *E. histolytica*.

In addition to the inductions of the genes discussed above, down-regulation of several genes encoding metabolic enzymes was also observed upon L-cysteine deprivation (Table 2; Additional file 3). L-Cysteine deprivation resulted in down-regulation of the expression of a gene encoding a novel NADPH-dependent oxido-reductase (EHI_045340). *E. histolytica *possesses two isotypes of these oxido-reductases (EhNO1 and 2) which contain FAD- and 2[4Fe-4S]-binding domains [34]. However, the expression of only EhNO2 *(*EHI_045340), but not of EhNO1, was dramatically down-regulated in a time-dependent manner upon deprivation of L-cysteine. This gene was also induced by 7 fold upon the supplementation of 10 mM of L-cysteine in to the culture medium for 48 h [34]. In contrast, the level of EhNO1 remained unchanged in either presence or absence of L-cysteine [34]. Our recent biochemical analysis showed that EhNO1 and 2 catalyse the NADPH-dependent reduction of oxygen to hydrogen peroxide, and L-cystine to L-cysteine, and also function as ferric and ferredoxin-NADP^+ ^reductases. EhNO2 possesses 4-fold higher L-cystine reduction efficiency than EhNO1, where as EhNO1 is more efficient in reducing ferredoxin and ferric ion [34].

L-Cysteine deprivation also led to the down regulation of two genes encoding dUTP nucleotidohydrolase, which convert dUTP to dUMP, and thus are involved in removing dUTP from the deoxynucleotide pool, reducing the probability of this nucleotide being mistakenly incorporated into DNA. Genes encoding aspartate aminotransferase and aspartate ammonia lyase which are involved in the catabolism of Glu, Asp, and Asn were down-regulated on L-cysteine deprivation (Additional file 3). These amino acids can be catabolised to pyruvate through malate and fumarate [35]. As a result of decreased utilization of pyruvate upon L-cysteine deprivation, malate and fumarate are accumulated. Down-regulation of aspartate aminotransferase, and aspartate ammonia lyase will, in theory, lead to the decreased catabolism of these amino acids, and will prevent further accumulation of malate and fumarate. Aspartate aminotransferase in various organisms is known possess L-cysteine aminotransferase activity, which leads to the formation of mercaptopyruvate from L-cysteine [36]. Like EhNO2, this enzyme might also be regulated by the availability of its alternative substrate (L-cysteine), which is highly decreased upon L-cysteine deprivation. We also noticed time-dependent modulation in the expression of genes encoding putative fatty acid elongases. It has been shown that L-cysteine depletion decreases PtdEtn, and thus affects PtdCho/PtdEtn ratio, which potentially alters membrane fluidity, integrity, protein translocation across membranes, and membrane fusion events [12,37-39]. Changes in the expression of fatty acid elongases may be associated with the modulation of fatty acid chains in the phospholipids to compensate for the physical changes induced by the decrement in PtdEtn upon L-cysteine deprivation.

### Effect of L-cysteine deprivation on nucleic acid metabolism

Expression of several genes encoding proteins with functions in DNA/RNA metabolism was significantly modulated upon L-cysteine deprivation. They include several genes encoding regulator of nonsense transcripts (RENT), which participate in the nonsense mediated decay (NMD) of mRNAs containing a frameshift or a nonsense mutation (Figure 5B). This surveillance system protects cells from the production of non-functional proteins by eliminating mutant mRNAs. In addition to RNA surveillance, NMD is also involved regulating the abundance of hundreds of naturally occurring mRNAs [40].

The *E. histolytica *genome database at AmoebaDB [6,41] contains 8 different entries that showed similarity to the RENTs from other higher eukaryotes. Based on the fact that some of these entries showed very high mutual sequence identities (85-95%), there are only 4 independent RENT genes in *E. histolytica*. All of the probe sets representing amebic RENTs showed a common pattern of expression during L-cysteine deprivation. They were induced during early time points of L-cysteine deprivation, and then down-regulated during the later time points (Figure 5B). Thus, like *Giardia lamblia*, the components of NMD pathway seem to be present and functional in *E. histolytica*. In *G. lamblia*, a large number of naturally occurring transcripts have been shown to be under the control of NMD [42]. However, the functionality, its targets, and role of NMD in the gene regulation of *E. histolytica *have not yet been demonstrated. Changes in the expression of RENTs suggest that some of the observed changes in the gene expression upon L-cysteine deprivation might be resulted from the corresponding changes in the NMD pathway of mRNA degradation.

Beside RENTs, several other genes with functions in nucleic acid metabolism were also modulated upon L-cysteine deprivation (Additional files 2 and 3). A gene encoding a putative zinc finger protein was induced at the early time points, and then repressed at the later time points. Other genes, such as DNA/RNA helicase, a myb-like transcription factor, and a putative DNA repair protein were down-regulated upon L-cysteine deprivation. In addition, two genes encoding putative high mobility group (HMG) box proteins were slightly down-regulated upon L-cysteine deprivation. These proteins are associated with chromatin, and are involved in various processes including transcription, replication, recombination, and DNA repair [43]. Recently, expression of a large number of genes has been demonstrated to be modulated by the over-expression of a HMGB1 protein in *E*. *histolytica *[44]. Other genes encoding a putative La ribonucleoprotein and a ribosomal protein S30 were also up-regulated on L-cysteine deprivation. These results showed that L-cysteine modulates several genes involved in transcriptional and posttranscriptional regulation of the gene expression.

### Effect of L-cysteine deprivation on signal transduction

A significant number of genes (27) encoding signalling proteins were modulated in response to L-cysteine deprivation. Of these modulated genes, 11 were up-regulated and 16 were down-regulated (Figure 1). They include several genes encoding key signalling proteins such as protein kinases, phosphatases, guanine nucleotide exchange factors (Ras-GEF), GTPases, and GTPase activating proteins (GAPs). Alterations in mRNA abundance of these key signalling genes upon L-cysteine deprivation suggest a significant cellular re-programming to cope up with the consequences of L-cysteine deprivation or to help trophozoites get adapted to low cysteine environment. Deprivation of amino acids, including L-cysteine, is known to activates an amino acid response (AAR) that alters cellular functions by regulating the expression of various genes using transcriptional and post-transcriptional mechanisms [18,45]. Activation of AAR leads to increased protein abundance of activating transcription factors, which in turn modulate the expression of genes containing AAR element (AARE) [18,45]. However, such an AAR was not induced in *E. histolytica*, as it appears to lack activating transcription factors. These results suggest that *E. histolytica *does not employ canonical pathways to cope with the amino acid deprivation, but may employ other novel strategies.

### Effect of L-cysteine deprivation on vesicular trafficking, cytoskeleton, and secretion

Response to changing environmental conditions by eukaryotic cells also includes modulation of protein degradation, targeting, transport to specific organelles, and secretion. Amino acid deprivation has been shown to regulate vesicular trafficking, secretion, exocytosis, and autophagy [46]. L-Cysteine limitation also modulates several proteins associated with these processes in *E. histolytica*. For example, four genes encoding putative cysteine proteases (EHI_123950, EHI_121160, EHI_160330, EHI_182260) were down-regulated in a time-dependent manner during L-cysteine deprivation (Table 2; Additional file 3). A gene encoding vacuolar protein sorting 26 (Vps26) was up-regulated during L-cysteine deprivation. In addition, several genes encoding guanine nucleotide exchange factors (Ras-GEF), GTPases, and GTPase activating proteins (GAPs) were also modulated in response to L-cysteine deprivation. Modulation of the genes encoding putative ankyrin and actin binding protein suggested that L-cysteine deprivation may affect cytoskeleton re-organization, mobility and vesicular trafficking.

### Miscellaneous

In addition to the modulation of above mentioned genes expression of several other transcripts was also changed upon L-cysteine deprivation. For example, a transcript for a putative heat shock protein 20 was induced 4-5 fold, and two WD40 domain-containing proteins were down-regulated 3-4 fold upon L-cysteine deprivation (Additional files 2 and 3). WD-repeat proteins are a large family found in almost all eukaryotes and implicated in a variety of cellular functions ranging from signal transduction and transcription regulation to cell cycle control. One of the common functions of most of the WD-repeat proteins is to coordinate multi-protein complex assemblies [47]. Several genes encoding leucine-rich repeat proteins were down-regulated 3-6 fold at early time points upon L-cysteine deprivation (Additional file 3). Leucine-rich repeats serve as recognition motifs for surface proteins in bacteria and eukaryotes.

### Repression of genes encoding ISF causes growth defects

**Figure 6:**
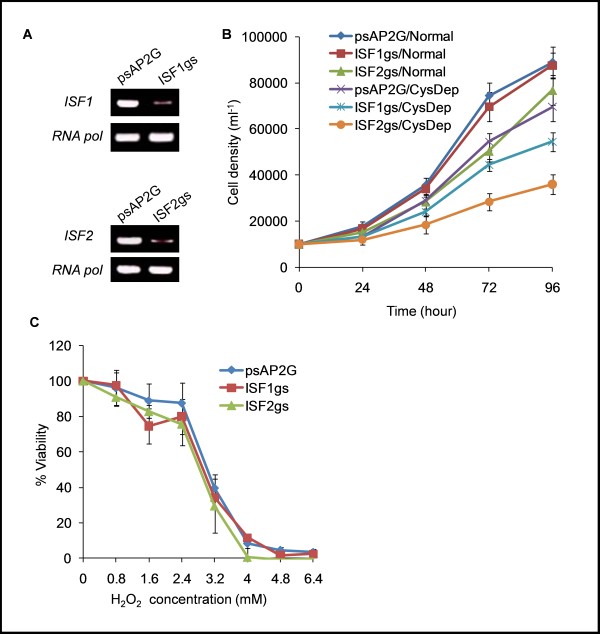
**Epigenetic repression of iron sulfur flavoproteins in *E. histolytica *G3 strain**. ***A***) Semi-quantitative RT-PCR analysis of *ISF1 *and *ISF2 *expression levels in G3 parasites transfected with either empty vector (psAP2Gunma) or gene silencing plasmids (psAP2G-*ISF1 *and psAP2G-*ISF2*). cDNA from these cell lines (psAP2G, *ISF*1gs, *ISF2*gs) were subjected to 30 cycles of PCR using specific primers for *ISF1 *or *ISF2*. RNA polymerase was used as a control. PCR from samples without RT served as controls to exclude the possibility of genomic DNA contamination. ***B***) Effect of *ISF1 *or *ISF2 *repression on the growth of trophozoites cultured under normal ("Normal") or L-cysteine-depleted ("CysDep") conditions. ***C***) Effect of *ISF1 or ISF2 *repression on the tolerance of amoebic trophozoites to oxidative stress.

In order to further characterize the functional role of the genes induced upon L-cysteine deprivation, we utilized the epigenetic silencing in *E. histolytica *G3 strain to repress genes of interest [48,49]. Using this epigenetic silencing strategy, we were able to repress (≥90%) two genes encoding ISFs (ISF1, EHI_138480 and ISF2, EHI_025710) that were highly induced gene upon L-cysteine deprivation (Figure 6A). However, we could not repress the third highly induced gene (MFS; EHI_173950). Repression of ISF2, but not of ISF1, showed slight growth deflects when cultured in normal medium. However, a severe growth defect in ISF2-repressed, and relatively mild growth defect in ISF1-repressed G3 trophozoites were observed in L-cysteine-deprived medium (Figure 6B). We also checked if repression of ISF1 or 2 also affects the tolerance of trophozoites to H_2_O_2 _mediated cytotoxicity. However, no significant difference in the tolerance to H_2_O_2 _cytotoxicity was observed (Figure 6C). L-Cysteine deprivation induced growth defects in ISF1- and 2-repressed G3 trophozoites suggest that in addition to their proposed roles in combating oxidative stress, ISF1 and 2 proteins may also play important roles under L-cysteine deprivation. These ISF are very similar to bacterial NADPH-dependent FMN reductases, which are induced upon sulfate or L-cysteine starvation [50]. In *Escherichia coli*, this enzyme, called a two-component alkanesulfonate monooxygenase, allows utilization of alkanesulfonates as sulfur sources under sulfate or cysteine starvation [29]. However, it still remains unclear whether ISFs in *Entamoeba *are also involved in similar processes.

## Conclusions

This study represents the first genome-wide analysis of transcriptional changes induced by L-cysteine deprivation in protozoan parasites, and in eukaryotic organisms where L-cysteine represents the major intracellular thiol. We showed global changes in the expression of genes implicated in metabolism, signalling, oxidative defence, DNA/RNA regulation, and transport. Although a large number of genes were modulated upon L-cysteine deprivation, significant transcriptional changes in genes involved in SAA metabolism were not observed, which confirmed that changes in the metabolic flux across SAA metabolism are not caused by the changes in the expression of corresponding genes. Similarly, we also showed that the changes in the gene expression induced by L-cysteine deprivation are not shared by those induced by oxidative or nitrosative stress. The most important changes that occurred upon L-cysteine deprivation were the induction of iron sulfur flavoproteins and major facilitator super-family transporter. Repression of ISF1 and 2 genes caused growth defects under L-cysteine-deprived conditions. Further studies on the kinetic and biochemical analysis of ISFs and MFS transporter, and their regulation should help to better understand the physiological role of these proteins in the biology of *E. histolytica*. L-Cysteine depletion mediated time-dependent changes in the expression of RENTs suggest that similar to other eukaryotic cells, NMD may also be functional in *E. histolytica*. This study also confirmed that most of the L-cysteine deprivation-mediated metabolomic changes in amino acid, central energy, and phospholipid metabolism are not associated with the changes in the expression of the corresponding genes. This general lack of correlation between metabolome, proteome, and transcriptome appears to be a general characteristic in various organisms including *E*. *histolytica*, indicating that they have more complex mechanisms of expression regulation.

## Methods

### Microorganism and cultivation

Trophozoites of the *E. histolytica *clonal strain HM1: IMSS cl 6 and G3 strain, kindly provided by David Mirelman, Weisman Institute, Israel [48,49], were maintained axenically in Diamond's BI-S-33 medium at 35.5°C as described previously [51,52]. Trophozoites were harvested in the late-logarithmic growth phase for 2-3 days after inoculation of one-thirtieth to one-twelfth of the total culture volume. After the cultures were chilled on ice for 5 min, trophozoites were collected by centrifugation at 500 × g for 10 min at 4°C and washed twice with ice-cold PBS, pH 7.4.

### RNA isolation and Affymetrix microarray hybridization

Trophozoites were first grown in normal culture medium containing a high concentration of L-cysteine (8 mM) for approximately 24 hrs. After culture medium was replaced with the one containing no exogenous L-cysteine, culture was continued for the next 3, 6, 12, 24, or 48 h. Total RNA was isolated from harvested trophozoites using Trizol reagent (Invitrogen, Carlsbad, CA, U.S.A.) according to the manufacturer's protocol. The RNA was quantified and checked for purity by comparison of absorbance at 260 and 280 nm in the NanoDrop Spectrophotometer (Thermo Scientific, Wilmington, DE, USA). Integrity of isolated RNA was verified by using Bio-Rad's automated electrophoresis system Experion (RNA StdSens analysis kit). All reagents and protocols followed those described in the Affymetrix manuals. Briefly, total RNA (5 μg) was reverse transcribed using T7-Oligo (dT) primer in the first strand cDNA synthesis. After second strand synthesis, the double-stranded cDNA template was used for in vitro transcription, in the presence of biotinylated nucleotides to produce labelled cRNA. The cRNA was purified, quantified, fragmented, and hybridized for 16 h at 45°C to custom-generated Affymetrix platform microarray (49-7875) with probe sets consisting of 11 probe pairs representing 9,327 *E. histolytica *(Eh_Eia520620F_Eh) and 12,385 *E. invadens *open reading frames (Eh_Eia520620F_Ei). After hybridization, the arrays were washed and stained with streptavidin-phycoerythrin using a GeneChip^® ^Fluidics Station 450 (Affymetrix, Santa Clara, CA, USA), according to the recommendations of the manufacturer. After washing and staining, the GeneChip^® ^arrays were then scanned using the Hewlett-Packard Affymetrix Scanner 3000 (Affymetrix, Santa Clara, CA, USA), and the probe intensities were extracted using Affymetrix^® ^GeneChip^® ^Command Console™(Affymetrix, Santa Clara, CA, USA).

### Analysis of microarray data

A minimum of two arrays were used for each condition and each time point. Raw Mas5 gene expression data were imported into the GeneSpring GX 10.0.2 program and normalized expression values for each probe set were obtained from raw probe intensities in R 2.7.0 (downloaded from the BioConductor project http://www.bioconductor.org) using robust multiarray averaging with correction for oligosequence (gcRMA). Standard correlation coefficients were calculated using GeneSpring GX 10.0.2. One way ANOVA analysis with Tukey's post hoc test was performed to extract differentially expressed genes. The p-values were calculated using Welch's test, and were corrected by Benjamini-Hochberg method.

### Quantitative real-time PCR

Total RNA from the trophozoites cultured in either normal or L-cysteine-deprived medium was extracted as described above. cDNA was synthesized from 5 μg of total RNA using Superscript III First-Strand Synthesis System, and oligo(dT)_20 _primer (Invitrogen). PCR was performed with the resulting cDNA as a template and specific oligonucleotide primers using the ABI PRISM 7300 Sequence Detection System (Applied Biosystems, Weiterstadt, Germany). A list of primers for qRT-PCR is shown in Additional file 6. Parameters for PCR were: an initial step of denaturation at 95°C for 9 min followed by 40 cycles of denaturation at 94°C for 30 s, annealing at 50°C for 30 s and extension at 65°C for 1 min. A final step at 95°C for 9 s, 60°C for 9 s and 95°C for 9 s was used to remove primer dimers [34].

### Creation of *E. histolytica *transformants where expression of the genes induced upon L-cysteine deprivation were repressed

In order to construct plasmids for epigenetic silencing of *ISF1*, *ISF2*, and *MFS*, a fragment corresponding to a 420-bp long 5' end of open reading frame of *ISF1*, *ISF2 *and *MFS *genes was amplified by PCR from cDNA using sense and antisense oligonucleotides containing *StuI *and *SacI *restriction sites, respectively. A list of these primers is provided in Additional file 7. These PCR amplified products were digested with *StuI *and *SacI*, and ligated into the *StuI- *and *SacI*-double digested psAP-2-Gunma shuttle vector.

psAP-2-Gunma was constructed as follows. 5'ap-a fragment were amplified from psAP-2 [48,49], as a template, using sense and antisense oligonucleotides containing appropriate restriction sites at the 5' end, with 5'-AGCTCTAGA**ccgcgg**CGGCTTGCTGCACCCTTTG-3' primer and 5'-CTCT*gagctc*GAGCTCGTTTAAaggcctCATGATTGTTTGTAAGATAT G-3' primers (*SacII, SacI, and StuI *restriction sites are shown by bold-, italicized-, or underlined-text). PCR product and psAP-2 vector were digested by *SacI *and *SacII*. Digested PCR product was ligated into psAP-2 to yield psAP-2-Gunma vector (psAP2G).

*StuI- *and *SacI*-digested PCR products corresponding to a 420-bp long 5' end of open reading frame of *ISF1*, *ISF2 *and *MFS *genes were ligated into psAP-2-Gunma to construct gene silencing plasmids of target genes (psAP2G-*ISF1*, psAP2G-*ISF2*, and psAP2G-*MFS*). The trophozoites of *G3 *strain were transformed with either empty vector or silencing plasmids by liposome-mediated transfection as previously described [11]. Transformants were initially selected in the presence of 1 μg/ml geneticin (Invitrogen), and the geneticin concentrations were gradually increased to 7 μg/mL during the subsequent two weeks prior to subjecting the transformants to analyses. The expression of the respective genes was confirmed by semi-quantitative RT-PCR as described previously [23]. These transformants were named as psAP2G (control) or -ISF1gs, ISF2gs, and MFSgs.

### Growth assay of *E. histolytica *trophozoites

Approximately 6 × 10^4 ^exponentially growing trophozoites of *E*. *histolytica *G3 strain transformed with psAP2G-*ISF1*, psAP2G-*ISF2*, or psAP2G (control) plasmid were inoculated in 6 ml of normal or L-cysteine-deprived BI-S-33 medium containing 7 μg/mL geneticin, and the parasites were counted every 24 h on a haemocytometer.

### Assay of hydrogen peroxide sensitivity

To examine sensitivity to H_2_O_2_, *E. histolytica *G3 trophozoites harbouring psAP2G-*ISF1*, psAP2G-*ISF2*, or psAP2G were seeded into a 96-well plate (10^4 ^trophozoites per well) in BI-S-33 medium containing 7 μg/mL geneticin and incubated for 12-16 h at 35.5°C. The cells were then exposed to varying concentrations (0.8-6.4 mM) of H_2_O_2 _for 1 h in the same culture medium. Following incubation, medium was removed and 100 μL pre-warmed Opti-MEM^® ^I (Invitrogen) containing 10% (v/v) Cell Proliferation Reagent WST-1 (Roche Diagnostics, Mannheim, Germany) was added. After 1 h of incubation at 35.5°C, the optical density at *A*_450 _was measured with that at *A*_595 _as a reference using a microplate reader (Model 550, Bio-Rad, Tokyo, Japan). The initial density and incubation period of the cultures were chosen to maintain the control trophozoites in the late-logarithmic growth phase throughout the experiment, and also to allow the measurement of optical density in the linear portion of the curves. The assays were performed 3 times in triplicate.

## Abbreviations

ISF: Iron sulfur flavoprotein; MFS: major facilitator super-family; SAA: Sulfur-containing amino acid.

## Authors' contributions

Conceived and designed the experiments: AH, GJ, DS, TN. Performed the experiments: AH, GJ, DS. Analyzed the data: AH, GJ, DS. Contributed reagents/materials/analysis tools: TN. Wrote the paper: AH, TN. All authors read and approved the final manuscript.

## Supplementary Material

Additional file 1 **All transcriptomic data analyzed in this study**. Normalized average raw data (signal intensity), their converted data (in log_2_), and present call (P, present; M, marginal; A, absent) of the duplicates of all the probe sets at 0, 3, 6, 12, 24, and 48 h of L-cysteine deprivation are shown. Fold changes of expression relative to 0 h, and up/down-regulation of expression, as well as p-value and corrected p-value of ANOVA are also shown. Click here for file

Additional file 2 **List of genes induced ≥3 fold at one or more time points upon L-cysteine deprivation**. Probe ID, corrected p-value by ANOVA, fold change and up/down-regulation, and normalized expression levels in log_2 _scale at each time point are shown. Locus ID, accession numbers, annotations, and other information related to GO term, InterProScan domains are shown. Click here for file

Additional file 3 **List of genes down-regulated ≥3 fold at one or more time points upon L-cysteine deprivation**. Probe ID, corrected p-value by ANOVA, fold change and up/down-regulation, and normalized expression levels in log_2 _scale at each time point are shown. Locus ID, accession numbers, annotations, and other information related to GO term, InterProScan domains are shown. Click here for file

Additional file 4 **List of changes in expression of genes that are involved in sulfur-containing amino acid metabolism upon L-cysteine deprivation**. Normalized average raw data (signal intensity), their converted data (in log_2_), and present call (P, present; M, marginal; A, absent) of the duplicates of all the probe sets at 0, 3, 6, 12, 24, and 48 h of L-cysteine deprivation are shown. Fold changes of expression relative to 0 h, and up/down-regulation of expression, as well as p-value and corrected p-value of ANOVA are also shown. Click here for file

Additional file 5 **List of 41 genes modulated ≥3 fold by L-cysteine deprivation and also modulated ≥3 fold by oxidative (1 mM of H_2_O_2 _for 1 h) and/or nitrosative stress (200 μM of DPTA-NONOate for 1 h)**. The list contains genes shown in Figure 3B. Click here for file

Additional file 6 **List of primers used for qRT-PCR**. Click here for file

Additional file 7 **List of primers used for the construction of plasmids for the repression of genes that were induced upon L-cysteine deprivation**. Click here for file
